# Removal of Ammonium Ions from Aqueous Solutions Using Weathered Halloysite

**DOI:** 10.3390/ma14164359

**Published:** 2021-08-04

**Authors:** Jacek Leszczyński

**Affiliations:** Department of Technology in Environmental Engineering, Faculty of Civil Engineering and Environmental Sciences, Bialystok University of Technology, 15-351 Białystok, Poland; j.leszczynski@pb.edu.pl

**Keywords:** weathered halloysite, ammonium ion, ion exchange, isotherm, kinetic model

## Abstract

This study investigated the use of weathered halloysite as an ion exchange material for ammonium removal from water. The study was conducted under static and dynamic conditions. The influence of such parameters as the preliminary concentration of ammonium ions, dose of halloysite, and pH was examined in periodic studies. The ion exchange capacity of weathered halloysite under various regeneration conditions such as concentration, excess of regeneration solution and the pH at which the regeneration was performed was also determined. The effect of flow velocity, initial NH_4_^+^-ions concentration was studied in column tests and the weathered halloysite’s ion -exchange capacity was also determined. The best results of ammonium ion removal were obtained at pH 6. The equilibrium isotherms were described using the Langmuir and Freundlich models. The results of periodic studies show a good fit for the data of both models, with Langmuir isotherms reflecting the removal of ammonium ions better. A good match for the data (R^2^ > 0.99) was provided by a pseudo second-order kinetic model. The obtained results indicate that a properly prepared halloysite can be a useful mineral for the removal of dangerous substances, such as ammonium ions, present in natural waters.

## 1. Introduction

Ammonium nitrogen water pollution is a real issue in many areas of the world. Ammonium ions can enter water, through communal and industrial wastewater and surface runoff from fields and meadows. Ammonium nitrogen in surface water accelerates eutrophication and is harmful to aquatic organisms. Excess ammonium in water may reduce the effectiveness of disinfection and leads to the formation of disinfected byproducts [1,2,3,4,5]. Several biological and physicochemical processes such as air stripping, ion exchange, nitrification, and reverse osmosis are used to remove ammonium ions from water solutions [2,6,7]. Due to ion exchange and sorption properties, natural aluminosilicates, mainly zeolites, such as clinoptilolite, sepiolite, mordenite, and bentonite are commonly used for water and wastewater treatment [2,8,9,10]. The main physicochemical properties of zeolites are high sorption capacity, ion exchange capacity, and chemical and thermal resistance. Zeolites, which are crystalline hydrated logged aluminosilicates, contain micropores and are well-known ion exchangers [2,11,12,13]. The skeleton of the zeolite consists of symmetrically arranged aluminium oxide and silica tetrahedron, creating a stable three-dimensional structure with a negative charge neutralised by positively charged cations such as sodium and potassium. These cations can be exchanged with some of the ions present in aqueous solutions, such as heavy metals and NH_4_^+^ ions [11,12,14]. The use of zeolites to remove ammonium ions, due to their properties and characteristics, has been widely discussed in many scientific studies worldwide [11,15,16]. Park et al. [17] achieved an 80% efficiency of NH_4_^+^ removal using clinoptilolite from an aqueous solution containing 80 mg/L. Clinoptilolite originating from New Zealand was also studied by Weatherley and Miladinovic [18]. The authors investigated zeolites’ ability to remove ammonia in the presence of calcium, magnesium, and potassium cations. Balci and Dincel [19], obtained a 60% ammonia removal efficiency with sepiolite. Sarioglu [20] examined the efficiency of NH_4_^+^ removal with zeolite from the Dogantepe region of Turkey. Sprynskyy et al. [21] investigated the removal of ammonium ions from synthetic aqueous solutions by raw and pre-processed natural Transcarpathian mordenite. The ion exchange capacity of mordenite relating to ammonium ions was estimated at 1.64 meq/g at an initial concentration of 1000 mg N-NH_4_^+^/L. Rozic et al. [22] examined the removal of NH_4_^+^ ions from aqueous solutions with Croatian bentonite and clinoptilolite. The maximum efficiency of NH_4_^+^ removal (61.1% by weight) was achieved at the initial concentration of 100 mg N-NH_4_^+^/L. By increasing the ammonium nitrogen concentration, the removal efficiency decreased rapidly [22]. Englert and Rubio [11] removed ammonia from water using natural Chilean zeolite composed mainly of clinoptilolite and mordenite of 1.02 meq NH_4_^+^/g ion exchange capacity, whereas Sprynskyy et al. [23] demonstrated clinoptilolite’s ability to remove heavy metals. Huang et al. [24] studied the adsorption of NH_4_^+^ on weathered crust elution-deposited rare earth ore (WCE-DREO) and clay minerals. The obtained adsorption capacities were as follows: montmorillonite > WCE-DREO > halloysite > illite > kaolinite. The J-type Linde zeolite, synthesised from raw kaolinite by Kamyab and Williams, performed well in the sorption of ammonium ions from aqueous solutions [25]. Mahata et al. [26] used Cu-loaded amine-functionalised SiO_2_ and a weakly basic ion exchange resin for NH_4_^+^ removal. In this study, the authors obtained a high adsorption capacity for the sorbent of 9.30–21.37 mg/g; the used adsorbent was successfully regenerated in 0.15 M NaCl solution.

Besides clinoptilolite, sepiolite, mordenite, and bentonite, halloysite is also used for water and sewage treatment [27]. This mineral occurs in many formations, particularly weathered volcanic rocks. Basalt spills containing large amounts of halloysite are found in Lower Silesia [28]. Halloysite is a clay mineral with a layered structure formed by SiO_4_ tetrahedral and AlO_6_ octahedral sheets. Halloysite has many industrial applications due to its well-developed specific, surface area [29,30]. It can be used as an adsorbent for treating liquids and process gases, for the production of nanocomposites, as an additive for wastewater treatment, as a catalyst in chemical processes, and as a multicomponent coagulant [27,31,32,33]. However, the available literature lacks comprehensive studies on the use of halloysite to remove NH_4_^+^ ions from aqueous solutions by ion exchange. There are currently three active mines for this resource globally, located in the USA, New Zealand, and Poland. One of the largest deposits of halloysite in the world is located in Poland’s Lower Silesia region [28,31,32,34].

This paper presents a new possibility for using weathered halloysite as an ion-exchange medium to remove NH_4_^+^ ions from aqueous solutions. Ammonium ion removal with the use of aluminosilicates is an important and current research issue. However, there has been no research on halloysite. Thus, in this work, filtration beds fabricated from weathered halloysite were created and applied to remove NH_4_^+^ ions from aqueous solutions. The ion exchange capacity, kinetics, and balance of NH_4_^+^ removal were studied, and factors controlling the process rate were determined. Moreover, the influence of coexisting cations such as Mg^2+^ and Ca^2+^ was studied, and various operational parameters necessary for effective regeneration of the mineral were tested. Due to the lack of available studies on halloysite, the results obtained were compared to clinoptilolite, sepiolite, mordenite, and bentonite.

## 2. Materials and Methods

### 2.1. Reagents

H_2_SO_4_ 95–97% (*w*/*w*), HCl, NaOH, and NH_4_Cl from Sigma-Aldrich, Poznań, Poland were used.

### 2.2. Characterization of Weathered Halloysite

The mineral used for this research came from Legnica in the Lower Silesia region of Poland. The mineral obtained in the region consists mainly of weathered tertiary halloysite of the kaolinite type. The main components of the samples are halloysite and kaolinite. Small amounts of montmorillonite, illite, anatase, and quartz [28] are also present. The chemical composition of weathered halloysite, in terms of oxides, is shown in Table 1.

The natural mineral was initially crushed and washed with distilled water, dried at 105 °C for 12 h, and then grinded to a size of less than 0.1 mm. The mineral was then mixed with distilled water and granulated. The pellets were heat-treated at 600 °C for 2 h. Processed weathered halloysite particles of 2.1–2.5 mm granulation were used. In order to convert the processed weathered halloysite into a sodium form, the granules were mixed with a 0.5 mol NaCl solution at pH 10.5 and shaken for 24 h, then washed with distilled water and finally dried at 60 °C. According to the studies, the regeneration of zeolites with alkaline NaCl solution increases regeneration efficiency and reduces the required volume of regeneration solution [35,36].

### 2.3. Chemical Analyses

Analytical tests of water samples included the following determinations: pH, alkalinity, conductivity, NH_4_^+^, Mg^2+^, Ca^2+^, Na^+^, and K^+^. All analyses were conducted according to the standard APHA procedure [37]. NH_4_^+^ concentration was determined by the standard Nessler method using a HACH DR 4000 spectrophotometer (Hach Lange GmbH, Düsseldorf, Germany). Calcium, magnesium, sodium, and potassium were determined by Atomic Absorption Spectrometry (AAS). The chemical composition of clay samples was determined with a TXRF (Total X-ray Reflection Fluorescence, S2 PICOFOX, Bruker, Germany).

Statistical analysis included the calculation of mean value and standard deviation. The presented test results are the mean of at least three repetitions, and the standard deviation of the measured values did not exceed 5%.

### 2.4. Experimental Procedures

The studies were conducted periodically under flow conditions using model water prepared from distilled and treated surface water (tap water). An aqueous solution of NH_4_^+^ in distilled and tap water was prepared from NH_4_Cl. The surface water came from the Supraśl River situated near the city of Białystok in Poland. The composition of the treated water is presented in Table 2.

#### 2.4.1. Batch Test

The effectiveness of ammonium ion removal was periodically tested depending on contact time, mineral dose, NH_4_^+^ concentration, and pH. The volume of solutions in individual experiments was 500 mL. The influence of initial NH_4_^+^ concentration was studied in the range of 5, 10, 15, 20, and 30 mg/L using a 15 g weathered halloysite balance. The removal efficiency of NH_4_^+^ depending on the dose of weathered halloysite was tested using 6, 9, 18, 27, and 36 g of mineral. The NH_4_^+^ removal efficiency depending on pH was tested in a range from 4 to 10 at a concentration of 20 mg NH_4_^+^/L and a weathered halloysite dose of 15 g. The effect of contact time was tested with an ammonium ion concentration in the range of 10 to 60 mg/L and a weathered halloysite dose of 5 g. During the experiment, at specific intervals, 5 mL samples were taken for analytical tests.

During the study, properly prepared doses of the regenerated mineral were introduced into prepared aqueous solutions and shaken for 45 min. The time was determined based on known research and literature. Dimova et al. [38] discovered that the uptake of NH_4_^+^ ions by aluminosilicate is a fast reaction, which occurs in less than 15 min. After shaking, the sample was filtered through a microporous membrane filter (0.45 µm), and the residual content of the NH_4_^+^ ion in the sample was tested. The concentration in the solid phase was calculated using the equation shown in Equation (1) [39]:

(1) $$q_{e}=\frac{\left(C_{o}-C_{e}\right)V}{M}$$

where $q_{e}$ (mg/g) is the total amount of adsorbed NH_4_^+^ ions (mg/g), C_e_ and C_o_ are the equilibrium and initial concentrations of NH_4_^+^ ions (mg/L), respectively. M is the adsorbent weight (g), and V is the solution volume (L).

#### 2.4.2. Regeneration

The influence of regeneration conditions on ion exchange capacity was tested at NH_4_^+^ 500 mg/L and a weathered halloysite dose of 5 g in 250 mL samples. Solutions of 3 and 5% NH_4_Cl were used for regeneration using the excess of three and five times the ion exchange volume. The tests were performed at pH levels of 9 and 10.5 of NH_4_Cl solution, determined with NaOH.

#### 2.4.3. Column Test

In the column test’s parameters, such as initial NH_4_^+^ concentration and flow rate, were examined. The influence of water hardness on the removal efficiency of NH_4_^+^ ions was also investigated, and the total and working ion exchange capacity of the weathered halloysite was determined. For the experiments, a glass filtration column with a bed volume of 67 mL and a diameter of 18 mm was used. The evaporated model water was fed by a peristaltic pump from top to bottom, keeping the water level in the column constant. The column was filled with granules with a diameter of 2.1–2.4 mm. Under flow conditions, the removal of ammonium ions was studied depending on the surface load of the filter column at ranges of 4, 6, 8, and 10 m/h and the initial concentration of NH_4_^+^ ions in the range of 5–50 mg/L. Exhaustion of the ion exchange bed was tested at a hydraulic load of 6 m/h. The test was conducted until breakthrough, and until the ion exchange capacity was completely exhausted, i.e., until the equilibrium concentration in the effluent was reached.

## 3. Results and Discussion

### 3.1. Batch Study

#### 3.1.1. Influence of Contact Time and Initial NH_4_^+^ Concentration

**Figure 1:**
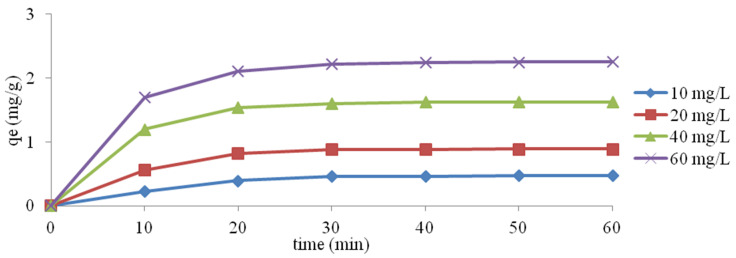
Influence of initial concentration and contact time on the exchange of NH_4_^+^ ions.

The efficiency of ion exchange depending on the reaction time and initial concentration of ammonium ions is shown in Figure 1. As illustrated in Figure 1, the removal rate of ammonium ions increases in the initial 20 min, then gradually equalises and finally reaches equilibrium after 40 min. Changes in the NH_4_^+^ removal rate in the initial phase of the process may result from many free active adsorbent sites. Then the removal rate of ammonium ions decreases. On the other hand, an increase in the ion exchange efficiency with an increasing initial NH_4_^+^ ion concentration may result from an increase in the driving force, which is measured by the concentration of the solution, especially in the initial stage of the process, where there is a large difference in the concentration of the ammonium ion [39,40,41,42,43].

#### 3.1.2. Influence of pH

**Figure 2:**
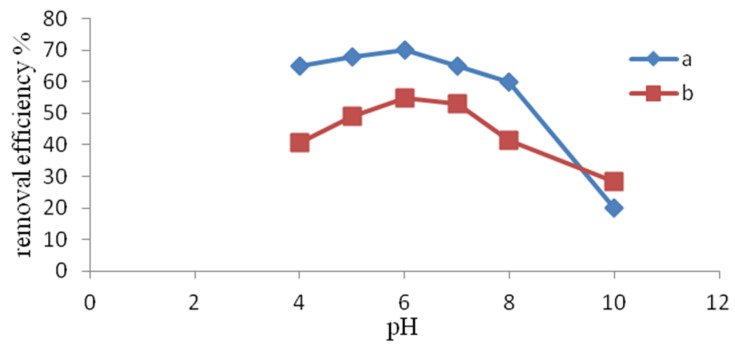
Influence of pH on the efficiency of NH_4_^+^ removal from (**a**) distilled water, (**b**) tap water.

The influence of pH in the studied range of 4–10 on the change in ammonium ion removal efficiency is shown in Figure 2. The highest reduction of NH_4_^+^ removal was obtained at a pH of 6. A slightly smaller effect was observed at pH 7. Similar properties were shown in clinoptilolite studied by Du et al. [35]. However, in these authors’ studies, a substantial decrease in the efficiency of the process was already noted at a pH of 7. The influence of the reaction on the efficiency of NH_4_^+^ ion removal is mainly related to the pH-dependent form of ammonium nitrogen. At a pH above 9, the NH_4_^+^ ions change into the gaseous form of NH_3_, which do not undergo ion exchange when deprived of charge. A drop in pH below 6 increases the concentration of H^+^ ions, which become competitive with NH_4_^+^ ions [41].

#### 3.1.3. Ammonium Exchange Isotherm

The removal of NH_4_^+^ ions by weathered halloysite was interpreted based on Langmuir and Freundlich isotherms. The isotherms show the dependence between the quantity of substance absorbed by the adsorbent mass units and adsorbate equilibrium concentrations. The models can be used to describe the behaviour of the ion exchange mass at equilibrium for various ammonium ion concentrations [20,44].

Freundlich’s isotherm is commonly used to characterise adsorption properties on heterogeneous surfaces. Freundlich’s equation usually takes the form of $q_{e}$ = KC_e_ 1/*n* ($q_{e}$ is the quantity of NH_4_^+^ absorbed per unit mass of adsorbent), C_e_ is the equilibrium concentration of NH_4_^+^ residual in solution, n and K are both empirical constants. The linear form of the equation is shown in Equation (2): [35,39]

(2) $${\mathrm{logq}}_{e}=\mathrm{logK}+\frac{1}{n}{\mathrm{logC}}_{e}$$

**Figure 3:**
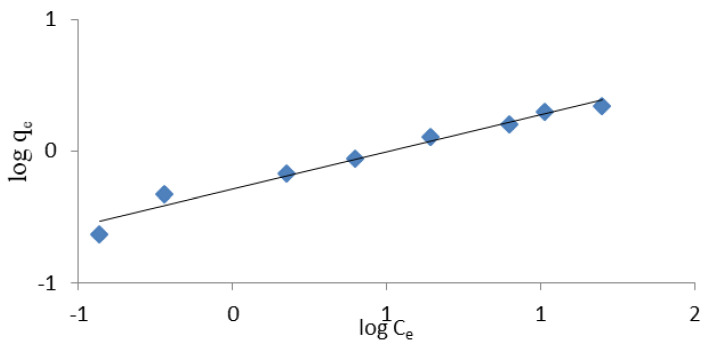
Freundlich linearised isotherm for the removal of ammonium ions from NH_4_Cl solution in distilled water.

**Figure 4:**
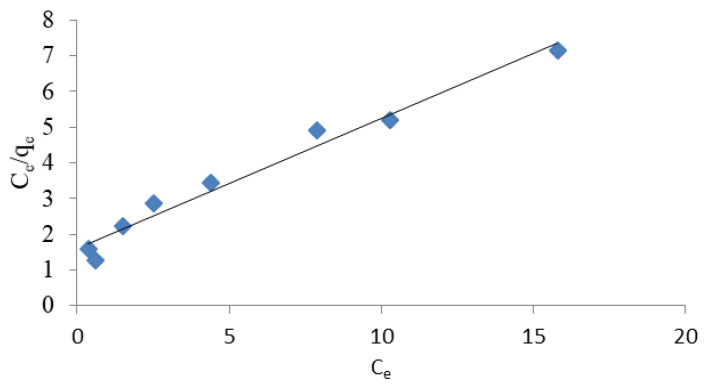
Langmuir linearised isotherm for the removal of ammonium ions from NH_4_Cl solution in distilled water.

**Figure 5:**
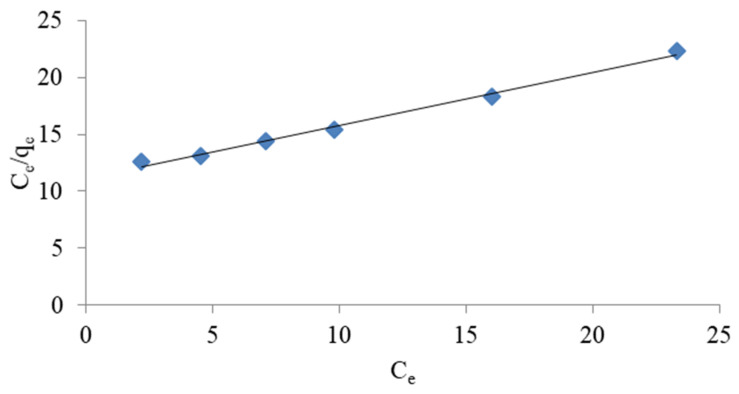
Langmuir linearised isotherm for the removal of ammonium ions from NH_4_Cl solution in tap water.

**Figure 6:**
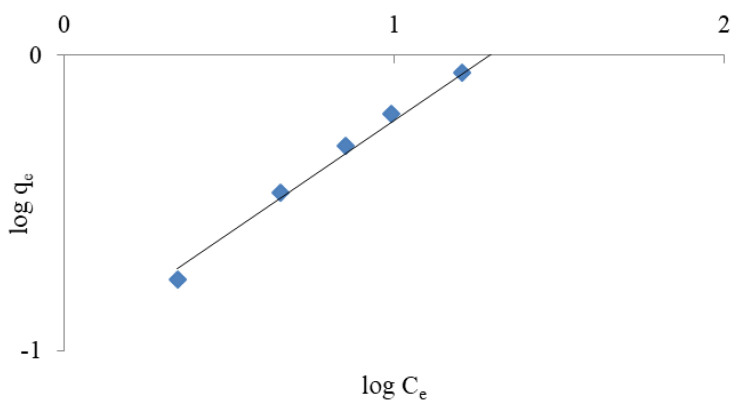
Freundlich linearised isotherm for the removal of ammonium ions from NH_4_Cl solution in tap water.

The Langmuir equation describes a case where only one adsorption layer is formed on the adsorbent surface. The Langmuir theory presumes that the adsorbent surface is homogeneous, and the possibility of creating multiple layers is excluded. A particle cannot move freely on the surface, and the lateral interactions between the particles are irrelevant [45,46,47]. The Langmuir equation in linear form is shown in Equation (3) [39]:

(3) $$\frac{C_{e}}{q_{e}}=\frac{1}{q_{m}K_{L}}\frac{C_{e}}{q_{m}}$$

where $q_{e}$ represents the amount of dissolved matter absorbed per unit weight of adsorbent in equilibrium (mg/g), parameter $q_{m}$ represents the maximum sorption capacity of the adsorbent, i.e., the maximum amount of ions needed to fill the monolayer (mg/g). C_e_ represents the equilibrium concentration of the substance dissolved in a solution (mg/L), and $K_{L}$ represents the Langmuir constant (L/mg). The $q_{m}$ and $K_{L}$ factors in the Langmuir equation are determined by the slope and intersection point of the graph Ce/$q_{e}$ to C_e_, respectively. However, the values of Freundlich K and Langmuir $K_{L}$ constants are a measurement of the relative adsorption capacity of NH_4_^+^ ions [18]. The experimental results shown in Figure 3, Figure 4, Figure 5 and Figure 6 indicate that both models fit well with the experimental data. The coefficients K, $K_{L}$, $q_{m}$ and (1/*n*), as well as the corresponding correlation factors for studies conducted with distilled and tap water, are presented in Table 3.

It can be seen that a good match was achieved for both models (R^2^ > 0.97). A better match for both models was specifically obtained in studies using tap water. Comparing the results obtained, however, it seems that the Langmuir isotherm better represents the adsorption capacity of halloysite and is more appropriate for use in ammonium ion removal studies. A better match of the Langmuir isotherm (R^2^ = 0.927 − 0.969) with Turkish clinoptilolite is also provided by Karadag et al. [43]. Zamri et al. [48] observed that Freundlich’s model was the best fit to remove NH_4_^+^ ions from ion exchange resin, while Langmuir’s model better reflected the removal of heavy metals.

#### 3.1.4. Kinetic Study

The study of kinetic parameters helps predict the rate of adsorption. Selected models of adsorption kinetics were used to study the kinetics of ammonium ion removal, namely the pseudo first-order, pseudo second-order, and intramolecular diffusion model. The pseudo first-order model equation is described as follows in Equation (4) [43]:

(4) $$\log\left(q_{e}-q_{t}\right)={\log q}_{e}-\frac{k_{1}}{2.303}t$$

The pseudo -first order reaction rate constant k_1_ (1/min) is shown in Table 4.

The linear form for the pseudo second-order equation is described as follows in Equation (5) [43]:

(5) $$\frac{t}{q_{t}}=\frac{1}{k_{2}q_{e}^{2}{}_{}}+\frac{1}{q_{e}}t$$

The pseudo-second order reaction rate, constant k_2_ (g/mg min), is shown in Table 4.

Intramolecular diffusion can be represented by Equation (6) [43]:

(6) $$q_{t}=k_{d}t^{0.5}+C$$

where $q_{t}$ is the absorbed substance in (mg/g min), k_d_ represents the intramolecular diffusion rate constant (mg/g min^1/2^), C is the intersection point.

**Figure 7:**
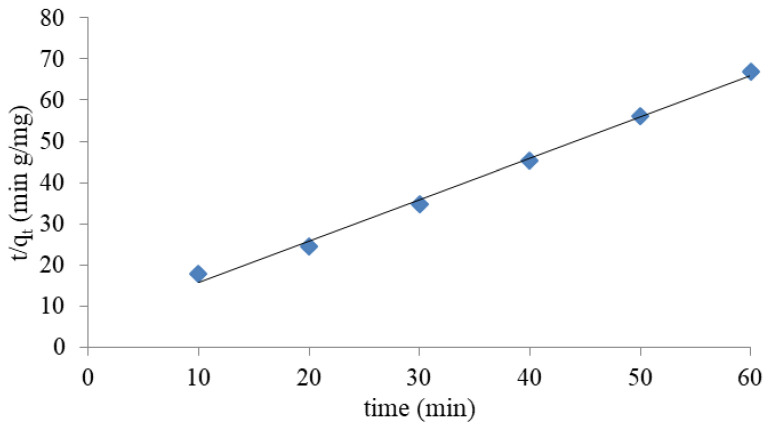
Second-order pseudo kinetic diagram for NH_4_^+^ exchange.

According to the study, a higher correlation coefficient (R^2^) was obtained for the pseudo second-order model and intramolecular diffusion. The data shown in Table 4 and Figure 7 demonstrated a fairly good fit (R^2^ > 0.99) with the pseudo second-order kinetic model; this indicates that this model describes the adsorption kinetics of NH_4_^+^ ions by halloysite. Therefore, this model is widely used to describe the kinetics of sorption and ion exchange processes and often provides the best fitting results [41,48,49].

**Figure 8:**
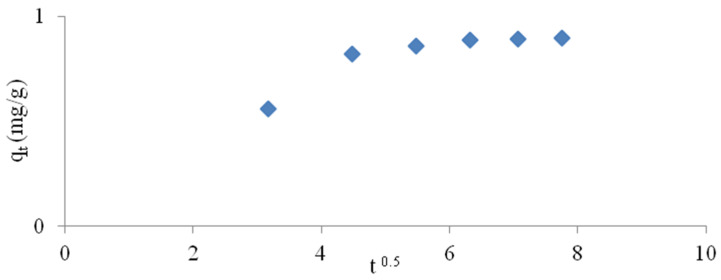
Intraparticle diffusion kinetic plot.

According to the intramolecular diffusion model, as shown in Figure 8, the ammonium exchange involves two stages (linear regions); this suggests that the process may be conducted by surface sorption and intramolecular diffusion [45]. Karadag et al. [43] have stated that the first linear part reflects the diffusion effect in the boundary layer, and the last linear part may be due to the intramolecular diffusion effect.

#### 3.1.5. Regeneration

**Figure 9:**
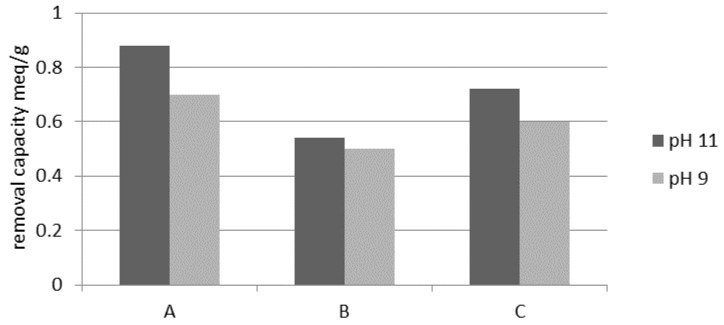
Weathered halloysite ion exchange capacity depending on pH excess and a concentration of the regenerating solution NaCl: A, 5% excess 5; B, 3% excess 3; C, 3% excess 5.

The results of weathered halloysite regeneration are presented in Figure 9. It was found that the highest ion exchange capacity relating to ammonium ions was obtained with 5% NaCl regeneration and a five-fold excess. The study also showed that apart from the NaCl concentration, the excess of regeneration solution is also an important parameter during regeneration. Regeneration with a NaCl solution of pH 10.5 significantly increased the ion exchange capacity of the mineral, especially when using a smaller excess of the regenerating agent. The obtained maximum ion exchange capacity of weathered halloysite (0.99 meq/g) is similar to that of clinoptilolite 1.03 mmol/g provided by Du et al. [35]. Sprynskyy et al. [21], on the other hand, evaluated the ion exchange capacity of Carpathian mordenite relating to ammonium ions at 1.64 meq/g at the initial N-NH_4_^+^ 1000 mg/L. Caradag et al. [43] obtained 6.32 mg N-NH_4_^+^/g for Turkish clinoptilolite. Widiastuti et al. [36] obtained 3.89 mg N-NH_4_^+^/g for Australian zeolite. Wang et al. [47] at NH_4_^+^ content of 10 ppm obtained 1.21 mmol/g for mordenite.

### 3.2. Column Study

**Figure 10:**
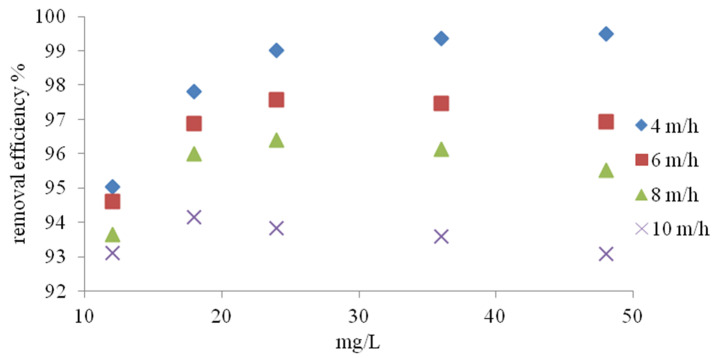
Effect of flow rate on the removal efficiency of different ammonia concentrations from NH_4_Cl solution in distilled water.

**Figure 11:**
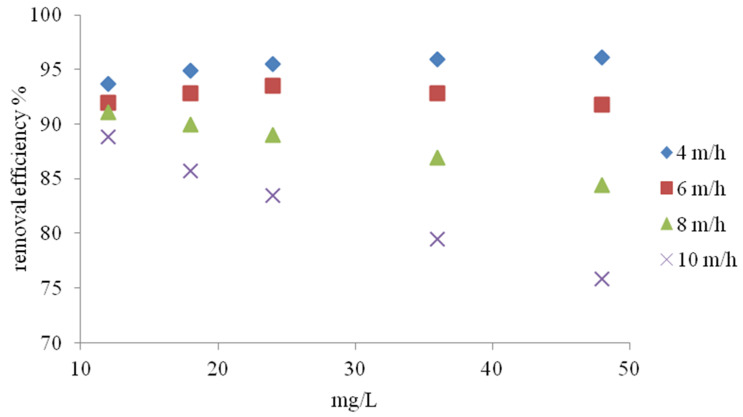
Effect of flow rate on the removal efficiency of different ammonia concentrations from NH_4_Cl solution in tap water.

Under flow conditions, the ion exchange efficiency was investigated depending on the filtration speed and initial concentration of ammonium ions. According to the studies presented in Figure 10, the efficiency of NH_4_^+^ ion removal depends on both the filtration speed and the initial concentration. In the case of filtration at a speed of 4 m/h, the effect of ammonium ion removal increased with the initial concentration, reaching 95.04% at 12 mg NH_4_^+^/L and 99.5% at 48 mg NH_4_^+^/L. During filtration at a speed of 6 and 8 m/h, the highest effect of NH_4_^+^ removal was obtained for the concentration of 24 mg NH_4_^+^/L. A decrease in process efficiency at higher ammonium ion concentrations was observed in both cases. At a filtration speed of 10 m/h, the maximum process efficiency was obtained with an initial concentration of 18 mg NH_4_^+^/L. Higher process efficiencies in the range of lower filtration rates are directly related to the retention time, which was also shown by Du et al. [35]. The results of tests using tap water are presented in Figure 11. At a filtration speed of 4 m/h, the process efficiency increased with the increase in initial NH_4_^+^ concentration from 93.1% to 96.1%. In the second case (filtration speed 6 m/h), the highest removal effect of 93.51% was obtained at the initial concentration of 24 mg NH_4_^+^/L. At the concentration of 48 mg NH_4_^+^/L, the process efficiency was lower and amounted to 91.8%. For the remaining filtration speeds, the effect of NH_4_^+^ ion removal decreased with increasing initial concentrations from 91.13% to 84.4% at 7 m/h and from 88.8 to 75.8% at 10 m/h. The decrease in ammonium ion removal efficiency in prepared tap water is mainly related to the presence of calcium and magnesium ions, which was confirmed by Transcarpathian clinoptilolite studies performed by Sprynskyy et al. [7] and Wang et al. [47]. Mazloomi et al. [40] showed the following ion exchange sequence (K > Na > Ca > Mg) for Iranian zeolites.

**Figure 12:**
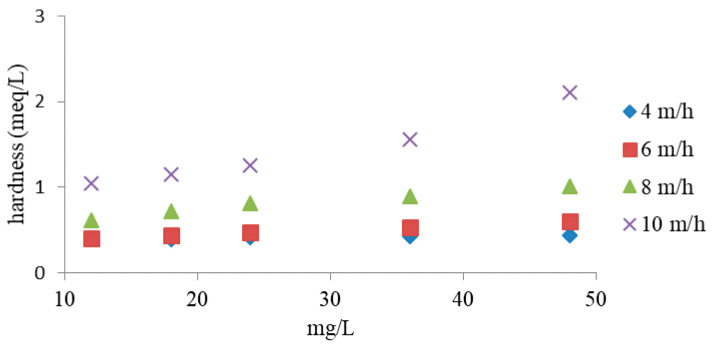
Relationship between initial ammonia concentration, hardness degree, and flow rate.

The relationship between the removal of Ca^2+^ and Mg^2+^ and NH_4_^+^ ions depending on the filtration rate is shown in Figure 12. According to the study, the removal efficiency of Ca^2+^ and Mg^2+^ decreased with the increasing filtration rate and initial NH_4_^+^ concentration. The obtained relationships indicate that weathered halloysite has a higher efficiency in removing ammonium ions than those causing water hardness at higher filtration speeds and higher initial NH_4_^+^ concentrations.

**Figure 13:**
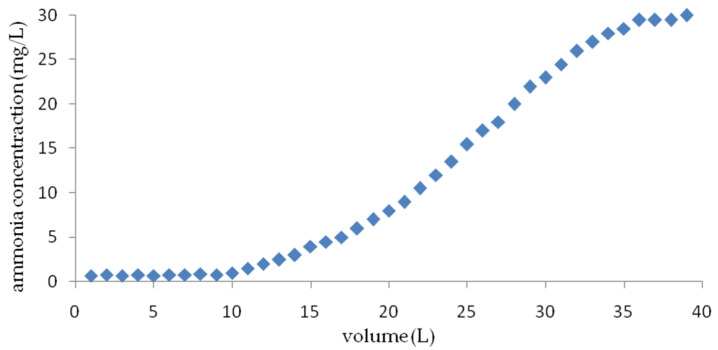
The breakthrough curve of ammonium ion removal by halloysite at an initial NH_4_^+^ ion concentration of 30 mg/L.

Figure 13 shows the exhaustion curve of an ion exchange column packed with a weathered halloysite at a flow rate of 6 m/h and an initial ammonium ion concentration of 30 mg NH_4_^+^/L. It was found that the total ion exchange capacity of the weathered halloysite obtained under dynamic conditions was 20 mg/g. The breakthrough of the halloysite bed was observed after passing 10 L of model water. The calculated ion exchange capacity was 5.0 mg/g. Du et al [35] determined that the CEC of clinoptilolite after reaching a concentration of 5 mg N-NH_4_^+^/L with an initial value of 25 mg N- NH_4_^+^/L, to be 7.74, 6.95 and 5.81 mg/g for flow rates of 6, 12 and 24 BV/h, respectively.

## 4. Conclusions

- The studies showed the usefulness of weathered halloysite to remove ammonium nitrogen from aqueous solutions. In addition, the results obtained confirm that properly prepared halloysite has a high ammonia removal efficiency, comparable to other aluminosilicates.
- In a periodic study, the effectiveness of NH_4_^+^ removal increased with the initial concentration, while the presence of hardness causing ions (Ca^2+^, Mg^2+^) in tap water decreased the effectiveness of the process.
- The removal of NH_4_^+^ also depended on the pH of the aqueous solution NH_4_Cl. The best result was obtained at a pH of 6, although a similar process efficiency was also recorded at pH 7. In the studied range of pH 4, 5, as well as 8, 9, the efficiencies of ammonium ion removal significantly decreased.
- Under dynamic conditions, the removal of NH_4_^+^ ions was closely related to the flow velocity and initial NH_4_^+^ concentration. In the study, it was observed that at a higher filtration speed and higher initial NH_4_^+^ concentration, weathered halloysite exhibited higher NH_4_^+^ removal efficiency than the ions causing water hardness.
- The experimental data described by the Langmuir and Freundlich isotherms provide the appropriate correlation coefficient value (R^2^ > 0.9). However, the Langmuir isotherm described the removal of ammonium ions by weathered halloysite slightly better.
- The pseudo second-order kinetic model provided the best correlation with experimental data for all systems tested.
- The weathered halloysite exchange capacity for NH_4_^+^ ions was evaluated as 0.99 meq/g at the initial NH_4_^+^ concentration of 500 mg/L. The highest capacity was obtained during weathered halloysite regeneration with a 5% NaCl solution using a five-fold excess. Apart from NaCl concentration, the excess of the regeneration solution was also an important parameter during the regeneration. Regeneration with a NaCl solution of pH 10.5 significantly increased the ion exchange capacity of the mineral, especially with a smaller excess of the regenerating agent.

## Figures and Tables

**Table 1:** Chemical characteristics of weathered halloysite.

Constituent	Value-wt.%
SiO_2_	35.74
Al_2_O_3_	24.09
Fe_2_O_3_	22.66
TiO_2_	3.92
CaO	1.24
MgO	0.61
Na_2_O	0.10
K_2_O	0.05
Loss of ignition (1000 °C)	6.64

**Table 2:** Characterization of tap water.

Parameter	Value
pH	7.4
Conductivity (µS/cm)	450
Alkalinity (mg CaCO_3_/L)	185.2
Total hardness (mg CaCO_3_/L)	246.3
KMnO_4_ (mg O_2_/L)	2.3
Ca^2+^ (mg/L)	76.5
Mg^2+^ (mg/L)	13.2
Na^+^ (mg/L)	9.6
K^+^ (mg/L)	1.3

**Table 3:** Langmuir and Freundlich model parameters.

Type of Sample	Freundlich			Langmuir		
	*K*	1/*n*	R^2^	*q*_m_ (mg/g)	*K*_L_ (L/mg)	R^2^
distilled water	0.51	0.57	0.971	2.75	0.226	0.974
tap water	0.11	0.76	0.987	2.139	0.042	0.993

**Table 4:** Pseudo first-order, pseudo second-order, and diffusion kinetic models.

Initial Concentration (mg NH_4_^+^/L)	Pseudo First-Order		Pseudo Second-Order		Diffusion
	k_1_ (1/min)	R^2^	k_2_ (g/mg min)	R^2^	R^2^
20	0.0599	0.91	0.19	0.99	0.73

## Data Availability

The data presented in this study are available on request from the corresponding author.
